# Adenoviral detection by recombinase polymerase amplification and vertical flow paper microarray

**DOI:** 10.1007/s00216-018-1503-y

**Published:** 2018-11-29

**Authors:** Susanna Nybond, Pedro Réu, Samuel Rhedin, Gustav Svedberg, Tobias Alfvén, Jesper Gantelius, Helene Andersson Svahn

**Affiliations:** 1 School of Engineering Sciences in Chemistry, Biotechnology and Health, Department of Protein Science, KTH Royal Institute of Technology, 100 44 Stockholm, Sweden; 2 Department of Medicine, Solna, Unit of Infectious Diseases, Karolinska Institutet and Karolinska University Hospital, 17176 Stockholm, Sweden; 3 Sachs’ Children & Youth Hospital, South General Hospital, 11883 Stockholm, Sweden; 4 Department of Public Health Sciences, Karolinska Institutet, 17177 Stockholm, Sweden

**Keywords:** Adenoviral, RPA, VFM

## Abstract

Respiratory viral infections often mimic the symptoms of infections caused by bacteria; however, restricted and targeted administration of antibiotics is needed to combat growing antimicrobial resistance. This is particularly relevant in low-income settings. In this work, we describe the use of isothermal amplification of viral DNA at 37 °C coupled to a paper-based vertical flow microarray (VFM) setup that utilizes a colorimetric detection of amplicons using functionalized gold nanoparticles. Two oligonucleotide probes, one in-house designed and one known adenoviral probe were tested and validated for microarray detection down to 50 nM using a synthetic target template. Furthermore, primers were shown to function in a recombinase polymerase amplification reaction using both synthetic template and viral DNA. As a proof-of-concept, we demonstrate adenoviral detection with four different adenoviral species associated with respiratory infections using the paper-based VFM format. The presented assay was validated with selected adenoviral species using the in-house probe, enabling detection at 1 ng of starting material with intra- and inter-assay %CV of ≤ 9% and ≤ 13%. Finally, we validate our overall method using clinical samples. Based on the results, the combination of recombinase polymerase amplification, paper microarray analysis, and nanoparticle-based colorimetric detection could thus be a useful strategy towards rapid and affordable multiplexed viral diagnostics.

**Electronic supplementary material:**

The online version of this article (10.1007/s00216-018-1503-y) contains supplementary material, which is available to authorized users.

## Introduction

Human adenovirus (HAdV) is associated with severe acute respiratory tract infections (ARTIs), together with other viruses such as influenza virus, human metapneumovirus, respiratory syncytial virus, and parainfluenza virus [1, 2]. ARTIs are common causes of hospitalization in children and can lead to severe illness and fatal outcomes [3, 4]. Rapid diagnosis of the etiological agent is needed in order to distinguish between viral and bacterial respiratory infections, which is of importance since the administration of unnecessary antibiotics during a viral infection contributes to growing antibiotic resistance [5, 6].

Traditional diagnostic assays such as virus culturing, serological assays detecting antibodies, or direct immunofluorescence of viral antigen have limited sensitivity and can be laborious and time-consuming [7]. Nucleic acid amplification techniques (NAATs) have started to replace traditional assays and current viral diagnostic assays are for the most part PCR-based and are usually performed in centralized hospital laboratories [7–9]. However, outbreaks of different epidemics of respiratory viruses have led to an increasing demand for rapid, near patient tests for various infectious diseases during recent years [10, 11]. An advantage of performing a rapid respiratory point-of-care (POC) test already at the primary or emergency care site would be to decrease turn-around time and improve the clinical management of febrile children [5]. While in some cases symptoms may guide a clinician towards a narrow selection of potential pathogens to test for, it is well known that in the case of upper respiratory infections, a host of viral and bacterial pathogens may give rise to the same range of unspecific manifestations, calling for panel-based diagnostics. Commercial options exist for panel-based POC NAATs [7], however at price ranges for instrumentation and individual tests that would prevent broad adaptation [12]. Thus, there is a great need for affordable multiplexed viral diagnostic assays that may also be adapted to near patient as well as low resource settings [10]. Desirable criteria for POC test detecting respiratory tract infections are for instance affordable, rapid (< 1 h), and allowing the possibility for multiplex detection [10]. The temperature of the amplification method is a factor that needs to be considered when aiming for a POC approach. Although portable PCR based assays have been developed with integrated heating devices [13], the cycling of high and low temperatures needed in a PCR-based assay are highly energy and time demanding activities. Therefore, assays based on isothermal amplification at a constant temperature appear well suited for amplification in POC testing [14]. A number of different isothermal amplifications methods have been introduced to date of which many have been shown to be applicable for viral and infectious disease detection [15]. Recombinase polymerase amplification (RPA) has recently gained popularity as a convenient isothermal NAAT for diagnostics [16]. With an amplification time of 20 min, RPA is among the fastest isothermal methods as well as having one the lowest operating temperatures (37–42 °C). Although there are other isothermal methods operating at similar temperatures, the assay times can be up to 1–2 h and may also need an initial temperature denaturation of 95 °C. At low amplification temperature, the specificity of the primer hybridization is decreased and can lead to increased non-specific primer hybridizations. Instead of the heat denaturation step, the RPA method uses a recombinase protein that forms a nucleoprotein filament with the primers that can identify homolog sequences in dsDNA. RPA can thus initiate and maintain amplification at low temperature with high primer specificity without specific requirements for primer melting temperatures [14–18]. Paper-based devices are typically used as inexpensive platforms for diagnostic assays, and one of the most commonly used paper-based assays formats is the lateral flow (LF) strip test. Paper-based nucleic acid biosensors based on lateral flow have been recently developed to detect PCR and isothermal amplification products for detection of different infectious disease agents including targets such as bacteria, parasites and viruses [19]. In the LF setup, the sample is added at the beginning of a paper-strip from where the analytes in the sample are transported by capillary forces along the paper-membrane until they are bound by capture molecules printed in the test line [20]. Detection by LF strip assays is usually limited to one target, although newer developed assays can include several targets in one strip. One of the main challenges in paper-based diagnostics tests still needing improvement is measurement of larger panel of targets simultaneously. In for example immunoassay-based LF strips multiplex detection of several targets can be hampered by antibody cross-reactivity to several targets which can lead to non-specific binding [21]. In DNA-based detection hybridization of target to capture oligonucleotides is specific, although cross-reactivity still can occur.

**Fig. 1:**
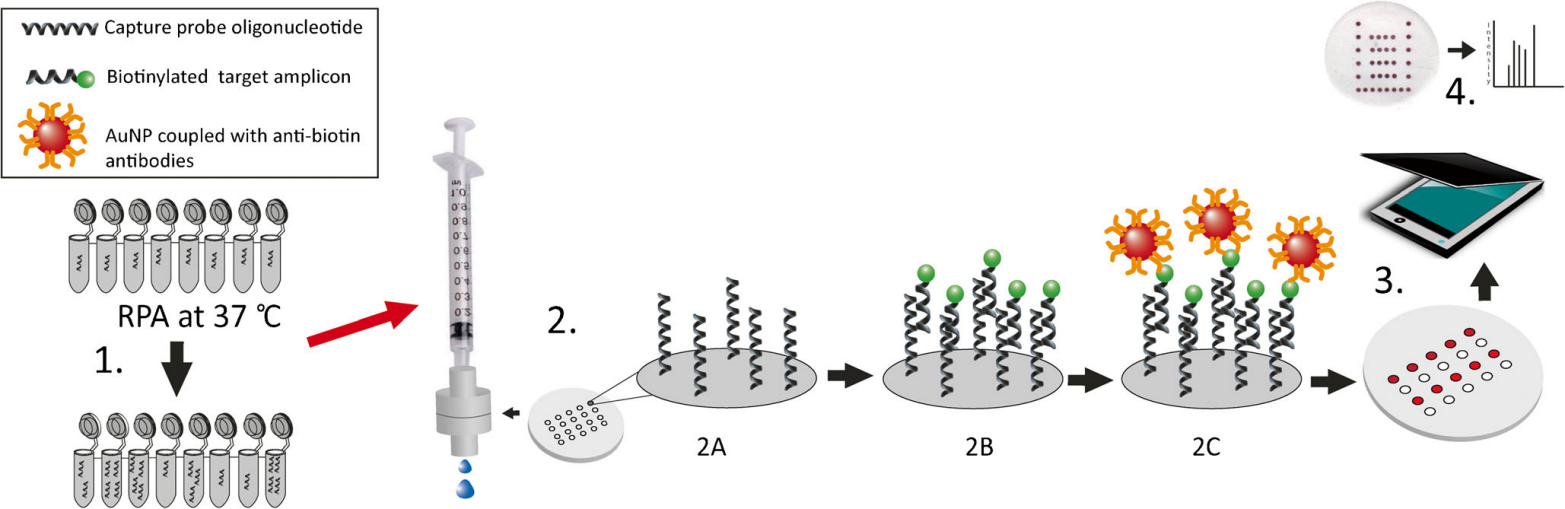
Viral detection using vertical flow microarray. In step 1, viral DNA is amplified and made single stranded. Step 2, **A** A nitrocellulose membrane where capture probes have been pre-printed is inserted into the VFM holder. **B** When the sample is flown vertically through the membrane, the biotinylated target amplicons anneal to the capture probes. **C** Positive spots are visualized by colorimetric detection by gold nanoparticles coupled to anti-biotin antibodies. In steps 3 and 4, the array membrane is scanned and the spot intensities are analyzed

By using microarray-based detection, multiplex detection of different oligonucleotide targets can be achieved simultaneously and DNA microarrays have been used for multiplex diagnostic purposes [22–24]. However, conventional printed nucleic acid microarrays have for the most part been implemented using glass slide microarrays [22]. Conventional microarrays protocols are also implemented using long incubation times of several hours due to slow diffusion of analytes to the capture probes [23, 24]. In a DNA microarray, probe-target hybridization can also be hampered by limitations of analyte transfer especially in static microarray systems lacking stirring or pumping for active movement of analytes [25]. Microarray detection using a LF strip can present several challenges such as evaporation of fluid or changes in flow speed along the strip. Reagent depletion and/or alteration of sample by upstream targets can lead to lower spot intensity of downstream spots [26]. Therefore, microarrays based on active vertical flow (VF) of analytes can be of advantage. In a VF setup, the sample is flown perpendicularly towards and through the paper sensor surface (Fig. 1). Analytes are thus actively transported by convection to the site of the capture probes, usually driven by an outer force such as or a pump, vacuum or centrifuge, but can also be executed by capillary forces using absorbent pads under the membrane [27, 28].

Although many paper-based microarrays work sufficiently well without any signal enhancement, there are cases where an additional signal enhancement step is necessary in order to achieve the required sensitivity and limit of detection. Care must be taken to consider the limitations of a low-resource environment when designing a signal enhancement strategy; the use of enzymes for example can be problematic due the limited stability of enzymes when stored at RT [29]. We have previously developed a signal enhancement strategy based on gold deposition that is rapid, enzyme-free, and uses reagents that can be stored for long periods of time at RT, making it potentially suitable in a POC context [30]. Here, we demonstrate the applicability of this method for increasing signal-to-noise ratios in paper-based detection of adenovirus DNA.

We have previously developed a paper-based protein microarray using a vertical flow microarray (VFM) format with colorimetric detection using antibody conjugated gold nanoparticles (AuNPs) [27]. The use of VFM was shown to have a potential for greater multiplexing capability when compared to lateral flow-based assays and could therefore be a better option for multiplexed POC tests [27]. In this work, we demonstrate amplification of adenoviral DNA with RPA and show the utility of the novel combination of VFM in paper-based colorimetric DNA microarray format for use as a detection technique for viral DNA amplified with RPA, using adenoviral detection as a proof-of-principle.

## Materials and methods

### Probes and primers

Adenoviral hexon gene sequences were retrieved from GenBank by a BLAST analysis. A consensus sequence of the amplicon was determined with the Jalview software by alignment of the amplicon sequence obtained from selected GenBank entries from different HAdV species: A (AB330093.1), B (JF713001.1), C (KP732095.1), D (KR150664.1), E (JF712986.1). An in-house capture probe was designed for the consensus sequence with Picky software and the selected capture probe was also checked using Mfold software for any possible self-folding properties*.*

The primers and probes used in this work (Table 1), as well as a synthetic version of consensus amplicon sequence (132 bp), were obtained from Biomers (Germany). All synthetic oligonucleotides were dissolved and stored in TE buffer at 100 μM (Thermo Fisher Scientific, USA); fresh dilutions were prepared for each RPA experiment into nuclease-free water. The sense (+) strand of the synthetic template contained a biotin-TEG 5′ modification as well as the sense primer, while the antisense primer contained a 5′ phosphate end. The probes were modified with a C6 amino-link at the 5′ end.

**Table 1:** Primers and probes used in this work

Oligo name	Sequence (5′-3′)	5’-Modification
HAdV sense primer*	GCCCCAGTGGTCTTACATGCACATC	Biotin-TEG
HAdV antisense primer*	GCCACGGTGGGGTTTCTAAACTT	Phosphate
HAdV probe 1	TGAAGTAGGTGTCTGTGGCGCGGGCGAACT	Aminolink C6
HAdV probe 2*	TGCACCAGACCCGGGCTCAGGTACTCCGA	Aminolink C6

*References [32, 33]

### Probe spotting and membrane blocking

The capture probes were spotted onto nitrocellulose membranes (Amersham, Protran 0.1 μm*,* GE Healthcare Life Science, Germany) using a Nano-Plotter 2 piezoelectric robotic printer (GeSiM, Germany). The probes were printed in PBS complimented with 1.5 M betaine (Sigma-Aldrich Chemie, Germany). During the initial tests, the probes were printed at different probe concentrations, 5, 10, 20, and 40 μM, spotted with 5 droplets per spot. Later on, specific probe concentrations were selected and the printing adapted to 10 droplets per spot. The microarray layout also consisted of a grid of visual positive control spots obtained by printing any DNA oligonucleotide with a biotin end-modification. The humidity during probe printing was kept at around 40% by a humidifier. The printed membranes were first dried for a few hours and then blocked with a 3% bovine serum albumin (BSA) solution for 20 min with 100 rpm agitation. BSA was discarded and the membranes washed twice for 15 min with 5 mM phosphate buffer and dried overnight.

### RPA

The amplification using RPA was performed with the TwistAmp Basic kit (TwistDx Ltd., Cambridge, UK) according to the manufacturer. Briefly, the sense and antisense primers are dissolved to 10 μM in nuclease free water of which 2.4 μL of each primer solution is added to one lyophilized pellet which is also hydrated with kit specific rehydration buffer and water. Subsequently, the template was added (either synthetic dsDNA template or standard adenoviral DNA in nuclease free water), yielding the total reaction volume up to 47.5 μL. The RPA reaction was started by addition of 2.5 μL 280 mM magnesium acetate. The standard incubation time of 20 min was employed by using a water bath at 37 °C.

### Purification and ssDNA generation

After RPA, the amplicon product was purified using the MinElute Reaction Cleanup Kit (Qiagen) according to manufacturer’s instructions. The purified amplicon products were diluted to a volume of 50 μL in elution buffer. During assay development, 5 μL of the dsDNA product was used for verification of amplicons by agarose gel electrophoresis (2% agarose stained with gelred). Before VFM, the dsDNA amplicons were generated to single-stranded biotinylated amplicons using lambda exonuclease (Thermo Fisher Scientific, USA), which selectively digest the 5′-phosphorylated strand. The digestion was performed according to manufacturer’s instructions with an incubation time of 30 min.

### VFM

The AuNPs used were 40-nm particles conjugated with monoclonal anti-biotin antibodies (BBI solutions, England). The target sample (either synthetic template or purified amplicon products from RPA) was further diluted to 500 μL into 1 × TBS (pH 7.5). Similarly, the AuNPs were diluted 1:4 to a final volume of 500 μL in 1 × TBS. A schematic illustration of the assay setup is shown in Fig. 1. A round cutout of the array from the membrane slide was placed into a Swinnex 13-mm polypropylene filter holder (Sterlitech, USA). The target and the washing buffer (1 × TBS complemented with 0.05% Tween-20) were added vertically through the membrane using a syringe pump (PHD 2000, Harvard apparatus). First, 1 mL of washing buffer was run through (1 mL min^−1^), followed by the target sample (0.45 mL, 150 μL min^−1^), followed by a wash (1 mL min^−1^). Subsequently, the detection solution containing AuNP conjugate was added (0.45 mL, 1 mL min^−1^), followed by a final 1 mL wash step (1 mL min^−1^). The array was then dried for 5–10 min before scanning with a consumer-grade flatbed scanner CanoScan 9000F Mark II (Canon), as a 16-bit grayscale TIFF file. For analytical purposes, the picture was then inverted and the spot intensity was analyzed using GenePix Pro 5.0 microarray analysis software (Molecular Devices, USA). The raw intensities obtained were the median intensity of each spot, which was then normalized with median background intensity of printing buffer spots (signal-background) and plotted using GraphPad Prism 7 software (GraphPad Software, Inc., USA).

### Viral DNA

Standard isolated adenoviral DNA was purchased from American Type Culture Collection (ATCC, USA/LGC Standards, Germany). The DNA acquired were total isolated DNA from infected cells, originating from four different adenoviruses; HAdV-31 (ATCC VR-1109), HAdV-3, (ATCC VR-847D), HAdV-1 (ATCC VR-1D), and HAdV-4 (ATCC VR-1572). Before RPA, DNA from each species was diluted in nuclease-free water; RPA was performed using 1 ng of DNA of each strain per reaction before amplification. The concentration of primers was kept the same as for previous experiments with synthetic template.

### Clinical samples

Nasopharyngeal aspirates were collected at Sachs’ Children and Youth Hospital, South General Hospital, Stockholm, Sweden, with ethical approval by the regional ethical committee in Stockholm (DNR: 2011/879-31/1). DNA was extracted with DNeasy Blood & Tissue Kit (69504 Qiagen) according to the manufacture. RPA was performed as described above and the products purified with PureLink Quick Gel Extraction & PCR Purification Combo Kit (K220001 Invitrogen) according to the manufacture. Finally, VFMs were printed with probe 1 at 40 μM and were run as described above.

### Signal enhancement

After imaging, the arrays were immersed in a signal enhancement solution consisting of 10 mM MES, pH 6 with 1 M H_2_O_2_ and 10 mM HAuCl_4_*3H_2_O. After 7 min, the arrays were removed from the solution, rinsed briefly in distilled water, and allowed to dry at RT. The dried arrays were then imaged one final time and image analysis was carried out using the same methodology as with the pre-enhanced arrays. Signal-to-noise ratio was calculated as the mean signal intensity from the replicate probe spits divided by the mean signal intensity from the replicate negative control spots.

## Results and discussion

### Validation of capture probes and colorimetric detection using synthetic template

The use of gold nanoparticles (AuNPs) enables detection as red color in the visible region, making AuNPs a suitable choice for colorimetric detection in diagnostic assays [31]. The use of colorimetric detection is especially useful in POC assays since it thus also enables visualization by naked eye or inexpensive optics without the need for advanced equipment such as an excitation source or equipment for signal emission quantification.

**Fig. 2:**
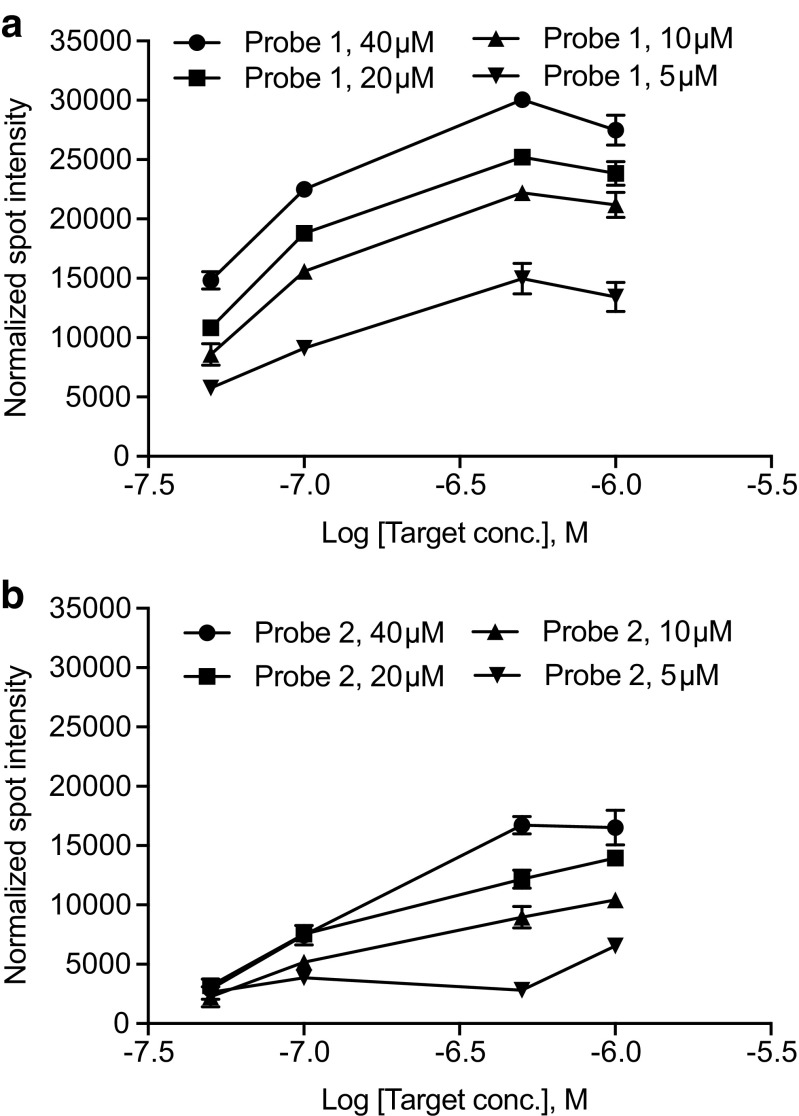
Evaluation of probe and target concentrations. VFM of the effect of different synthetic biotinylated sense strand target concentrations (1 μM, 500 nM, 100 nM, 50 nM). Probe 1 (**A**) and 2 (**B**) were printed on the array at different concentrations (5–40 μM). The error bars represent the SD of background normalized spot intensities of 3 replicate spots

In this work, two probes were chosen to be evaluated for functionality as capture probes on paper-based microarrays using colorimetric detection; an in-house designed probe (probe 1) and a second probe (probe 2) previously used in real-time PCR together with the pan-adenoviral primers [32]. The vertical flow setup was first validated by the direct use of the synthetic version of the amplicon template. Both capture probes showed increasing intensity with increasing probe concentration. The signal from both probes also displayed concentration dependency with target concentration. With excess target (1 μM), the signal of the hybridization of the template to the probes became saturated. The signal intensities of probe 1 were overall higher compared to probe 2 (Fig. 2). Probe 1 also showed high signal (above 10,000 units) even down to 50 nM target concentration, displaying 5 times higher normalized signal intensity at lowest target concentration compared to probe 2 (40 μM). Besides 50 nM, a lower target concentration of 10 nM was also run with VFM; however, neither of the probes showed any quantifiable signal at that target concentration.

### Validation of RPA using synthetic template

**Fig. 3:**
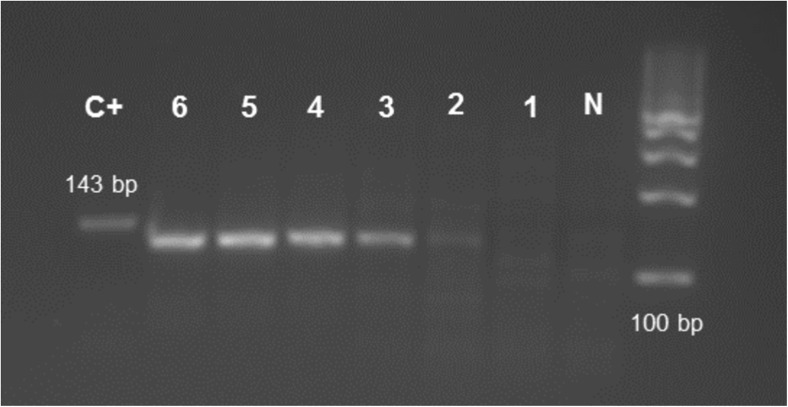
Validation of RPA amplification of synthetic template by gel electrophoresis. Lanes from ruler; N = RPA Negative (blank, only primer mix), Synthetic template: 1 = 22 copies, 2 = 216 copies, 3 = 2.16 × 10^3^ copies, 4 = 2.16 × 10^4^ copies, 5 = 2.16 × 10^5^ copies, 6 = 2.16 × 10^6^ copies. Copy numbers given are start copy numbers of synthetic target before RPA amplification. Target amplicon length = 132 bp. Absorbance of target dilution was at 260 nm was measured with Nanodrop and converted to template copy number. C+ = positive internal control included in RPA kit. Abbreviations: RPA, recombinase polymerase amplification

The gene sequence to be amplified for viral detection was selected in this work with the purpose of obtaining pan-adenoviral detection. Therefore, a primer pair for HAdV that amplifies a part of the hexon gene that has been shown to amplify human adenovirus in a pan-detection manner by PCR in clinical settings was chosen [32, 33]. The RPA kit used was the improved kit formulation, where also shorter PCR primers have been showed to function. RPA was also first performed by using the synthetic template, validating that the primers could successfully amplify the template in an isothermal amplification reaction. The synthetic dsDNA was diluted to different start copy numbers before RPA amplification. RPA amplicon products with a starting amount of ~ 2000 copies of template before amplification could be visualized after RPA by gel electrophoresis (Fig. 3, lane 3).

### Detection of synthetic template/RPA amplicons by vertical flow

**Fig. 4:**
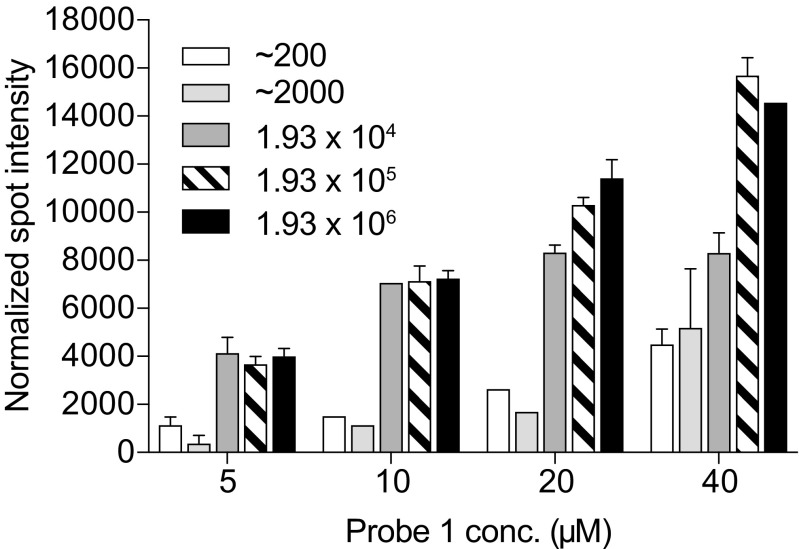
Validation of VFM setup. VFM using RPA amplicons originating from synthetic template at different start copy numbers before amplification. Abbreviations: RPA, recombinase polymerase amplification; VFM, vertical flow microarray

Next, VFM using the RPA amplicons generated from the synthetic template was performed (Fig. 4). The results are shown for probe 1; as at this stage, probe 2 did not generate high enough signals to be quantified. At very high starting amounts of template, probe 1 showed a quantifiable signal even at low print concentrations. At lower starting amounts of synthetic template, the concentration dependency of printed probe was higher. Although it was expected based on experiments using the pure synthetic template (Fig. 2) that the signals of probe 2 would be lower, it seemed that including the amplification and purification steps lowered the signal-to-background too low for probe 2. This effect could be seen in repeated experiments and throughout different probe print batches.

### Validation with adenoviral DNA

**Fig. 5:**
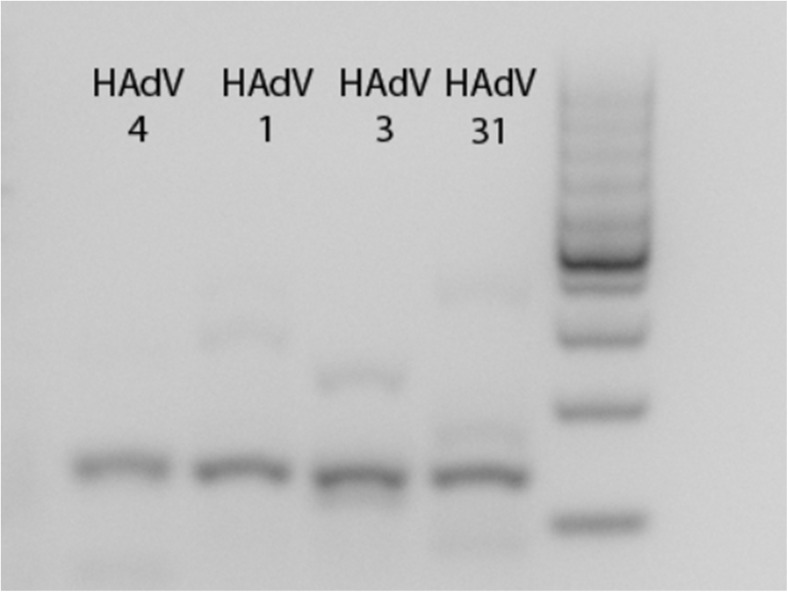
Recombinase polymerase amplification of adenoviral DNA. Start amount before RPA: 1 ng of ATCC standard viral DNA (total isolated DNA from infected cells). Abbreviations: RPA, recombinase polymerase amplification

**Fig. 6:**
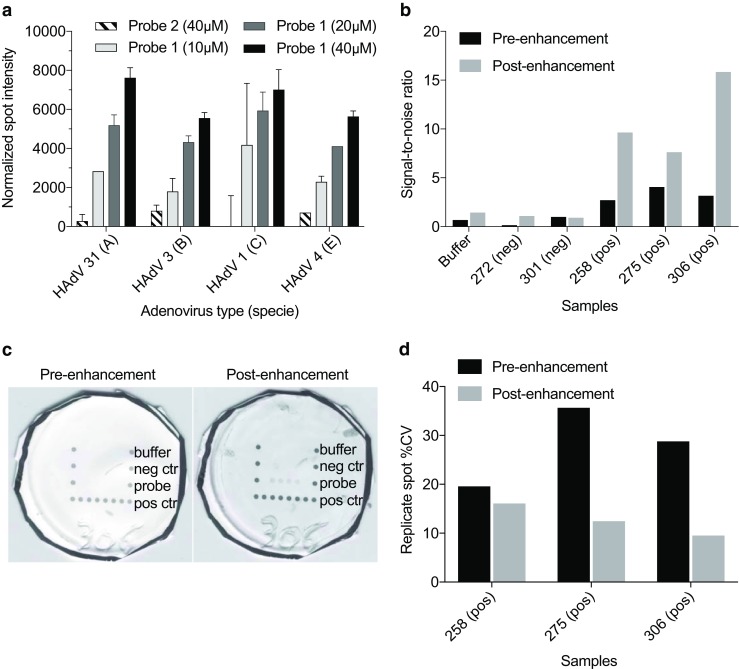
VFM of adenoviral amplicons after amplification. **A** Detection intensities of spots for the different adenoviral species at 3 different probe concentration for probe 1 and the highest concentration of probe 2. Start amount was 1 ng commercial viral DNA before amplification. Error bars indicate standard deviation of spot replicates (*n* = 4). **B** Comparison of signal-to-noise ratios measured from clinical samples before and after signal enhancement. **C** Scanned images of an array used to run a clinical sample (positive for adenovirus) before (left) and after (right) signal enhancement, demonstrating the improved visibility of the probe spots. **D** Signal enhancement lowers the variation between replicate spots

RPA and VFM were performed with commercial adenoviral DNA. There are seven HAdV species, (A to G), which can further be divided into different serotypes. The species that are related to respiratory infections are mainly A, B, C, and E types, while D, F, and G types are mostly associated with gastrointestinal infections and keratoconjunctivitis [34]. Thus, for this work, DNA from four adenoviruses representing a species with relevance to respiratory viruses (A, B, C, and E species) were amplified using RPA (Fig. 5). Vertical flow was then performed using the amplicons generated from adenoviral DNA (Fig. 6A). The results validated that different adenoviral species could be detected through RPA amplification and VFM using probe 1 (Fig. 6A). However, similarly to results using the synthetic template, only the highest concentration of probe 2 generated signal just above the background. Even though the second probe seemed to be less sensitive than probe 1 in a microarray setting, including a set of probes that combines detection with high and low sensitivity could potentially increase the detection range.

Amplicons from two of the adenoviruses (HAdV-31 and HAdV-1) were selected for further investigations of assay variation as the probes generated high array signal intensities using the highest concentrations of probe 1. Based on the results, using the probe at 40 μM showed intra-assay variation ≤ 9% between replicates and day-to-day variations ≤ 13%. Using probe 1 at lower printing concentrations of 20 μM and 10 μM resulted in 2–4 times higher %CV (Table 2). The specificity of probes was also validated by running a VFM on an unrelated amplicon product as a representative of a negative sample from another in-house project on arrays with the adenoviral probes, showing no signal from probes (see Electronic Supplementary Material (ESM) Fig. S1).

**Table 2:** Within assay and day-to-day variation of adenoviral detection for probe 1 at different spotting concentrations

Adenovirus*	Intra-assay (%CV)		
	40 μM	20 μM	10 μM
HAdV-31	3.1	8.4	6.5
HAdV-1	9.0	18.9	35.5
	Inter-assay (%CV)		
HAdV-31	12.2	20.0	20.7
HAdV-1	13.2	22.4	29.0

*Adenoviral DNA start material 1 ng before RPA. Intra-assay %CV is the mean of the three intra-assay CV (each assay contained 4 spot replicates). Inter-assay % CV was derived from three independent assays performed on different days

A final validation was performed using nasopharyngeal aspirates collected and pre-analyzed in the clinic (Fig. 6B, C). Similar to the buffer, the two negative samples had no signal, while the three positive samples revealed a measurable signal (Fig. 6B). After signal enhancement, up to a fivefold increase in signal-to-noise ratio was achieved in measurements on clinical samples (Fig. 6B). This corresponds to a large increase in the visibility of the probe spots (Fig. 6C), facilitating naked eye detection of spots that were previously too faint to see without imaging equipment and image analysis software. Adding the signal enhancement step can thus allow the assay to be run even in situations where such tools are not readily available. Additionally, the coefficient of variation (%CV) between replicate probe spots was decreased in the positive clinical samples following enhancement (Fig. 6D), increasing the reliability of the data. Although it has not been demonstrated here, enhancing the signal intensity could also potentially lead to an improvement in the sensitivity of the assay.

## Conclusions

We employed detection of human adenovirus as a proof-of-concept study to demonstrate the use of recombinase polymerase amplification combined with vertical flow microarray for rapid paper-based detection of a respiratory virus. By using our in-house designed pan-adenoviral capture probe, we validated that this setup could detect adenoviral DNA from four different adenoviral species with low intra- and inter-assay variation.

The use of paper-based vertical flow has several advantages compared to a lateral flow assay. With a lab-in-a-syringe design, VF provides a more controlled, targeted, and faster flow of analytes to the capture probes and enables more flexibility in adaptation to different assay formats as washing steps can easily be included. Moreover, the VF microarray format allows high multiplexing by inclusion of detection probes for multiple targets as individual spots on the membrane, enabling the possibility of a simple and rapid platform for multi-viral detection.

Multiplexed NAATs using several primer pairs in the same reaction mix is widely used in cases where target panels are small, and with the presented setup the amplification product from a multiplexed NAAT could be run directly on the VFM. If a multiplexed NAAT is inconvenient or impossible due to large target panels or primer pairs with high tendency for cross-reactivity [14], several parallel reactions could be performed followed by pooling to a final product mix that is subsequently run on the VFM.

Here, microarray detection was employed in pan-detection of adenovirus with a future aim to include a panel of capture probes specific to different respiratory viruses on one microarray. With this setup, a clinical sample could be run in a VFM format to serve as a first indication of the causing agent in a respiratory infection. Additionally, if needed, genotyping of different viral species could also be adapted to this setup by using species-specific capture probes.

In this work, we carried out RPA reaction at 37 °C by using a water bath incubation. Although water baths might not be available in resource-limited settings, RPA has also been tested to function using body heat [35], and thus could be easily adapted to different environments. Future work towards improved POC applicability of the VF assay includes replacing the pump with a portable pumping device and shifting to smartphone-based image acquisition and data analysis, allowing a higher degree of portability and off-grid use. Signal detection using VFM can be performed in < 10 min and the overall time for the method is currently ~ 1 h including amplification using RPA and sample preparative steps. Further optimization and shortening of assay conditions by simplifying the pre-VF sample preparations will decrease the overall assay time. Potential added steps for signal amplification could be considered for enhanced sensitivity. Future work also entails incorporating other respiratory viruses in the detection panel and further characterization of the method such as limit-of-detection studies using clinical patient samples.

## Electronic supplementary material

ESM 1 (PDF 142 kb)

## References

[1] Rhedin S, Lindstrand A, Hjelmgren A, Ryd-Rinder M, Ohrmalm L, Tolfvenstam T (2015). Respiratory viruses associated with community-acquired pneumonia in children: matched case-control study. Thorax.

[2] Pavia AT (2011). Viral infections of the lower respiratory tract: old viruses, new viruses, and the role of diagnosis. Clin Infect Dis.

[3] Liu L, Oza S, Hogan D, Perin J, Rudan I, Lawn JE, et al. Global, regional, and national causes of child mortality in 2000-13, with projections to inform post-2015 priorities: an updated systematic analysis. Lancet 2015;385(9966):430–440. 10.1016/S0140-6736(14)61698-6 25280870

[4] Tregoning JS, Schwarze J (2010). Respiratory viral infections in infants: causes, clinical symptoms, virology, and immunology. Clin Microbiol Rev.

[5] Thornton HV, Hay AD, Redmond NM, Turnbull SL, Christensen H, Peters TJ (2017). Throat swabs in children with respiratory tract infection: associations with clinical presentation and potential targets for point-of-care testing. Fam Pract.

[6] Lim YW, Steinhoff M, Girosi F, Holtzman D, Campbell H, Boer R (2006). Reducing the global burden of acute lower respiratory infections in children: the contribution of new diagnostics. Nature.

[7] Mahony JB, Petrich A, Smieja M (2011). Molecular diagnosis of respiratory virus infections. Crit Rev Clin Lab Sci.

[8] Rhedin S, Lindstrand A, Rotzen-Ostlund M, Tolfvenstam T, Ohrmalm L, Rinder MR (2014). Clinical utility of PCR for common viruses in acute respiratory illness. Pediatrics.

[9] Olofsson S, Brittain-Long R, Andersson LM, Westin J, Lindh M (2011). PCR for detection of respiratory viruses: seasonal variations of virus infections. Expert Rev Anti-Infect Ther.

[10] Zumla A, Al-Tawfiq JA, Enne VI, Kidd M, Drosten C, Breuer J (2014). Rapid point of care diagnostic tests for viral and bacterial respiratory tract infections--needs, advances, and future prospects. Lancet Infect Dis.

[11] Caliendo AM, Gilbert DN, Ginocchio CC, Hanson KE, May L, Quinn TC (2013). Better tests, better care: improved diagnostics for infectious diseases. Clin Infect Dis.

[12] Quinn AD, Dixon D, Meenan BJ (2016). Barriers to hospital-based clinical adoption of point-of-care testing (POCT): a systematic narrative review. Crit Rev Clin Lab Sci.

[13] Zarei M (2017). Portable biosensing devices for point-of-care diagnostics: recent developments and applications. TrAC Trends Anal Chem.

[14] Craw P, Balachandran W (2012). Isothermal nucleic acid amplification technologies for point-of-care diagnostics: a critical review. Lab Chip.

[15] de Paz HD, Brotons P, Munoz-Almagro C (2014). Molecular isothermal techniques for combating infectious diseases: towards low-cost point-of-care diagnostics. Expert Rev Mol Diagn.

[16] Daher RK, Stewart G, Boissinot M, Bergeron MG (2016). Recombinase polymerase amplification for diagnostic applications. Clin Chem.

[17] James A, Macdonald J (2015). Recombinase polymerase amplification: emergence as a critical molecular technology for rapid, low-resource diagnostics. Expert Rev Mol Diagn.

[18] Maffert P, Reverchon S, Nasser W, Rozand C, Abaibou H (2017). New nucleic acid testing devices to diagnose infectious diseases in resource-limited settings. Eur J Clin Microbiol Infect Dis.

[19] Rodriguez NM, Linnes JC, Fan A, Ellenson CK, Pollock NR, Klapperich CM (2015). Paper-based RNA extraction, in situ isothermal amplification, and lateral flow detection for low-cost, rapid diagnosis of influenza A (H1N1) from clinical specimens. Anal Chem.

[20] Posthuma-Trumpie GA, Korf J, van Amerongen A (2009). Lateral flow (immuno)assay: its strengths, weaknesses, opportunities and threats. A literature survey. Anal Bioanal Chem.

[21] Mohd Hanafiah K, Arifin N, Bustami Y, Noordin R, Garcia M, Anderson D. Development of multiplexed infectious disease lateral flow assays: challenges and opportunities. Diagnostics (Basel). 2017;7(3). 10.3390/diagnostics7030051 PMC5617951 28880218

[22] Miller MB, Tang YW (2009). Basic concepts of microarrays and potential applications in clinical microbiology. Clin Microbiol Rev.

[23] Martinez MA, Soto-Del Rio Mde L, Gutierrez RM, Chiu CY, Greninger AL, Contreras JF (2015). DNA microarray for detection of gastrointestinal viruses. J Clin Microbiol.

[24] Vora GJ, Meador CE, Stenger DA, Andreadis JD (2004). Nucleic acid amplification strategies for DNA microarray-based pathogen detection. Appl Environ Microbiol.

[25] Steger D, Berry D, Haider S, Horn M, Wagner M, Stocker R (2011). Systematic spatial bias in DNA microarray hybridization is caused by probe spot position-dependent variability in lateral diffusion. PLoS One.

[26] A. van Amerongen JV, H.A. Arends and M. Koets. Handbook of Immunoassay Technologies. 1 ed. Luong SKVaJHT, editor: Academic Press; 2018. 496 p.

[27] Chinnasamy T, Segerink LI, Nystrand M, Gantelius J, Andersson SH (2014). Point-of-care vertical flow allergen microarray assay: proof of concept. Clin Chem.

[28] Vella SJ, Beattie P, Cademartiri R, Laromaine A, Martinez AW, Phillips ST (2012). Measuring markers of liver function using a micropatterned paper device designed for blood from a fingerstick. Anal Chem.

[29] Quesada-Gonzalez D, Merkoci A (2015). Nanoparticle-based lateral flow biosensors. Biosens Bioelectron.

[30] Dias JT, Svedberg G, Nystrand M, Andersson-Svahn H, Gantelius J. Rapid nanoprobe signal enhancement by in situ gold nanoparticle synthesis. J Vis Exp. 2018;133. 10.3791/57297 PMC5931481 29578517

[31] Cordeiro M, Ferreira Carlos F, Pedrosa P, Lopez A, Baptista PV. Gold nanoparticles for diagnostics: advances towards points of care. Diagnostics (Basel). 2016;6(4). 10.3390/diagnostics6040043 PMC5192518 27879660

[32] Heim A, Ebnet C, Harste G, Pring-Akerblom P (2003). Rapid and quantitative detection of human adenovirus DNA by real-time PCR. J Med Virol.

[33] Tiveljung-Lindell A, Rotzen-Ostlund M, Gupta S, Ullstrand R, Grillner L, Zweygberg-Wirgart B (2009). Development and implementation of a molecular diagnostic platform for daily rapid detection of 15 respiratory viruses. J Med Virol.

[34] Ghebremedhin B (2014). Human adenovirus: viral pathogen with increasing importance. Eur J Microbiol Immunol (Bp).

[35] Crannell ZA, Rohrman B, Richards-Kortum R (2014). Equipment-free incubation of recombinase polymerase amplification reactions using body heat. PLoS One.

