# Supplementary material for: Adenoviral detection by recombinase polymerase amplification and vertical flow paper microarray
Source: Anal Bioanal Chem. 2018 Nov 29;411(4):813–22. doi: 10.1007/s00216-018-1503-y (PMC6338793; doi:10.1007/s00216-018-1503-y)
Supplement: Supplementary file 1 — (PDF 142 kb) [file 216_2018_1503_MOESM1_ESM.pdf]

Analytical and Bioanalytical Chemistry

Electronic Supplementary Material

Adenoviral detection by recombinase polymerase amplification and vertical flow paper microarray

Susanna Nybond, Pedro Réu, Samuel Rhedin, Gustav Svedberg, Tobias Alfvén,
Jesper Gantelius, Helene Andersson Svahn

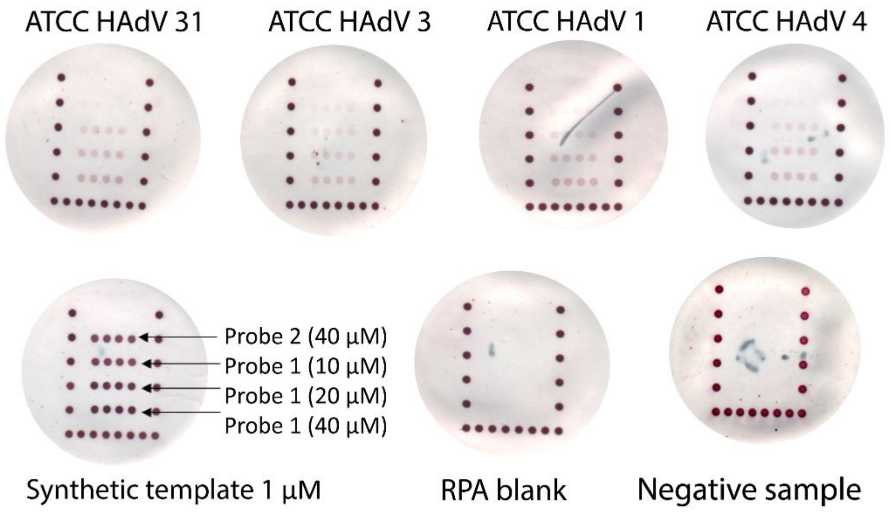

Fig. S1 Scanned color images of vertical flow microarrays. Arrays shown are; excess synthetic template for visualization of all capture probes, the results with adenoviral DNA and two assay negatives (RPA blank and unrelated amplicon target)
